# Rapid Photolysis of Gaseous Organic Nitrates Formed from Hydroxyl and Nitrate Radical Oxidation of α‑Pinene and β‑Pinene

**DOI:** 10.1021/acsestair.5c00183

**Published:** 2025-10-07

**Authors:** Masayuki Takeuchi, Yuchen Wang, Nga L. Ng

**Affiliations:** † School of Civil and Environmental Engineering, 1372 Georgia Institute of Technology, Atlanta, Georgia 30332, United States; ‡ College of Environmental Science and Engineering, Hunan University, Changsha, Hunan 410082, China; § School of Chemical and Bimolecular Engineering, Georgia Institute of Technology, Atlanta, Georgia 30332, United States; ∥ School of Earth and Atmospheric Sciences, Georgia Institute of Technology, Atlanta, Georgia 30332, United States

**Keywords:** atmospheric chemistry, photolysis, atmospheric fate, gas-phase kinetics, monoterpenes, organic nitrates, environmental chamber experiments

## Abstract

Photolysis of gaseous organic nitrates is crucial for understanding the formation and fate of air pollutants such as nitrogen oxides (NO_*x*_) and ozone (O_3_). Monoterpenes are prevalent biogenic volatile organic compounds (VOCs), greatly contributing to the formation of organic nitrates; however, there is currently a lack of experimental constraints on the photolysis chemistry of monoterpene organic nitrates. Here, we investigated the photolysis of monoterpene organic nitrates via novel dual chamber experiments, in which a large suite of organic nitrates was formed from hydroxyl and nitrate radical oxidation of α-pinene and β-pinene in one chamber and were introduced into another chamber to study photolysis. We directly measured their photolysis rates with chemical ionization mass spectrometry by minimizing the interferences of other types of chemical and/or physical reactions. The chamber photolysis rate constants vary depending not only on the molecular formulas of organic nitrates but also on the VOC type and oxidation condition. While the photolysis rate constants of 53.1% of the C_10_ organic nitrates for which the rate constants were estimated are on the order of 1 × 10^–5^ s^–1^ or larger, the other C_10_ organic nitrates exhibit little to no decrease in their measured signals. This highlights how differences in chemical structures can affect the photolability of organic nitrates. The most photolabile organic nitrate is C_10_H_17_NO_5_ (either hydroxy carbonyl nitrate or hydroperoxy nitrate) formed from nitrate radical oxidation of α-pinene and β-pinene, with the chamber photolysis rate constant of 1.1 (±0.1) and 1.3 (±0.3) × 10^–4^ s^–1^, respectively. When extrapolated to ambient conditions (solar zenith angle of 28.14° in summer), the photolysis rate constant is as large as 6.4 (±3.0) × 10^–4^ s^–1^ (corresponding to a photolysis lifetime of 0.43 ± 0.20 h). Compared to other loss processes (i.e., OH oxidation and dry deposition) of gaseous organic nitrates formed from nitrate radical oxidation of β-pinene, photolysis serves as either a comparable or dominant sink depending on the molecular formulas of organic nitrates. These findings have important atmospheric implications regarding the role of monoterpene organic nitrates in the spatial distribution of NO_*x*_ and O_3_ formation.

## Graphical abstract

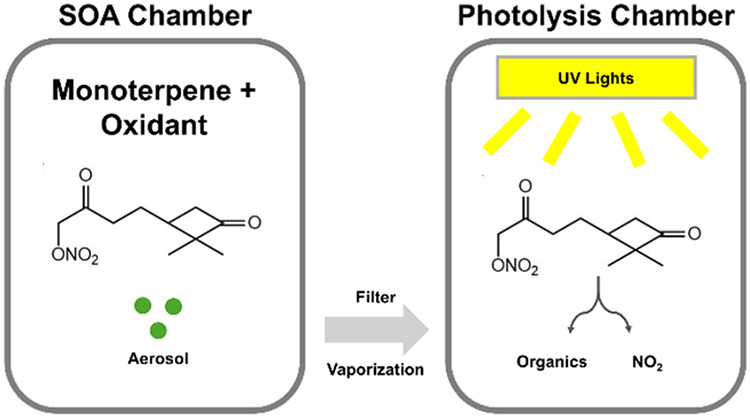

## Introduction

Organic nitrates make up an important class of atmospheric constituents. Formed from hydroxyl (OH) radical oxidation in the presence of nitrogen oxides (NO_*x*_) and from nitrate (NO_3_) radical oxidation of volatile organic compounds (VOCs), organic nitrates are reactive oxidized nitrogen species accounting for a significant portion of the atmospheric reactive nitrogen budget.^1,2^ They have been shown to play an important role in the regional and global distribution of NO_*x*_ via transport, affecting the formation potential of secondary air pollutants, such as ozone and secondary organic aerosol (SOA).^3^ To what extent organic nitrates affect the formation of secondary air pollutants depends on whether they act as a temporary reservoir or a permanent sink of NO_*x*_. Thus, it is important to evaluate the chemistry related to the fate of atmospherically relevant organic nitrates to better understand their roles in the secondary pollutant formation.

Monoterpenes (C_10_H_16_) constitute a major class of biogenic VOCs (∼15%) with annual emissions of 162 Tg yr^–1^.^4^ Organic nitrates formed from monoterpenes are prevalent in areas with substantial interactions between biogenic and anthropogenic emissions and with large contributions of monoterpene oxidation products to SOA, such as the southeastern U.S.^5–8^ Monoterpene emissions are not strongly dependent on light, and approximately 30 to 40% of their emissions occur at night.^9^ Since monoterpenes are precursors of organic nitrates regardless of the time of the day (daytime and nighttime), it is important to understand the chemistry of monoterpene organic nitrates formed from both daytime (OH radical) and nighttime (NO_3_ radical) oxidation. In addition, a recent study^10^ suggests that anthropogenic sources of monoterpenes can be as large as 37% in summer and 77% in winter in the urban area (Atlanta, Georgia) of the southeastern U.S. This further highlights the important interactions of anthropogenically coemitted monoterpenes and NO_*x*_.

The fate of monoterpene organic nitrates and the extent to which they serve as a temporary reservoir or a permanent sink of NO_*x*_ are highly uncertain.^11^ Photolysis of gas-phase monoterpene organic nitrates is considered a potential loss process, where NO_2_ is released back to the atmosphere via cleavage of an O–N bond in the nitrate functional group, serving as a temporary reservoir of NO_*x*_. Previous photolysis studies focused on small, simple gaseous organic nitrates and found that nitrate functional groups are photolabile.^12–18^ A recent study leveraged synthetic monoterpene hydroxy nitrates standards and showed that photolysis is an important sink for the studied monoterpene nitrates.^19^ While previous studies have provided valuable insights into the photolysis of organic nitrates, several key knowledge gaps remain. First, monoterpene organic nitrates exist with a broad range of chemical structures. While studies using synthetic standards provide fundamental insights,^19^ a more comprehensive study for the whole family of monoterpene organic nitrates formed via oxidation is warranted. Second, many of the proposed monoterpene organic nitrates are often multifunctional and contain photolabile functional groups, such as carbonyl and hydroperoxide groups.^20–25^ Synergistic enhancement of photolysis frequency has been previously reported,^17^ and multifunctional organic nitrates formed from oxidation of monoterpenes are likely to be affected in the same manner. Therefore, photolysis studies of chemically diverse monoterpene organic nitrates are needed to better understand the role of photolysis as a potential loss mechanism.

In this study, we investigate the photolysis of gaseous organic nitrates formed from OH and NO_3_ radical oxidation of monoterpenes (α-pinene and β-pinene) via laboratory chamber experiments. A novel dual chamber approach is used to separate gaseous organic species, including organic nitrates, from aerosol and oxidants, which can otherwise interfere with the measurements. Chamber photolysis rate constants (*j*_Chmbr_) of individual gaseous organic nitrates are directly measured by a high-resolution time-of-flight chemical mass spectrometer (HR-ToF-CIMS) using iodide as a reagent ion. The chamber photolysis rate constants are extrapolated to the ambient condition (solar zenith angle of 28.14° in summer) using the average ratio of ambient to chamber photolysis rate constants of synthetic hydroxy nitrates reported in our previous study.^19^ Chemical mechanisms of photolysis degradation are proposed for organic nitrates with previously proposed chemical structures. Finally, we evaluate how photolysis rates of monoterpene organic nitrates compare to other loss processes, such as OH radical oxidation and dry deposition.

## Methods

### Chamber Experiments

Laboratory chamber experiments were conducted at 22 °C under dry conditions (RH < 5%) at the Georgia Tech Environmental Chamber (GTEC) facility, housing two 12 m^3^ climate-controlled Teflon chambers surrounded by banks of UV lights (Sylvania 24922).^20^ The irradiation spectrum of the UV light was measured by a spectroradiometer (StellaNet Inc.) and was confirmed by a good agreement between the calculated and measured *j*_NO2_.

In a typical chamber experiment, the airborne constituents are very complex; organic compounds are present in both gas and particle phases, along with varying abundances of oxidants that are constantly transforming the chemical properties of the airborne constituents. This complex environment in the typical chamber experiment makes it challenging to study the effects and rates of isolated processes, such as photolysis.^26^ We addressed this by devising a novel dual chamber approach separating chamber-generated oxidized gaseous organic compounds from other constituents, such as inorganic gases, organic aerosol, inorganic seed particles, and oxidants. This procedure allowed us to directly investigate the photolysis of gas-phase organic nitrates, free from the interference of aerosol and oxidants and other processes (changing gas/particle partitioning, aerosol chemistry, etc.).

Prior to the experiments, both chambers were flushed with zero air (Aadco, 747–14) for at least a day and a half. In one chamber (SOA chamber), we generated SOA (on the order of milligrams m^–3^) from either OH radical or NO_3_ radical oxidation of VOCs. This high concentration of SOA allowed a substantial amount of semivolatile organic compounds to partition into the particle phase, which otherwise predominantly remained only in the gas phase. We followed the same methods used in our previous study to generate SOA.^27^ Briefly, precursor VOCs were α-pinene (99%, Sigma-Aldrich) and β-pinene (99%, Sigma-Aldrich). OH radicals were generated via photolysis of H_2_O_2_, while NO_3_ radicals were generated via thermal decomposition of N_2_O_5_, which was premade in a flow tube by simultaneously injecting 500 ppm of NO_2_ (Matheson) at 0.4 L min^–1^ and ∼250 ppm of O_3_ (Jelight 610) at 0.5 L min^–1^. Table S1 summarizes the initial concentrations of VOC and oxidants used to generate SOA. No seed aerosol was used in this study. Chamber relative humidity was under 5%, and the temperature in the reactors was kept at ∼22 °C.

The SOA formed in the SOA chamber was collected onto a Teflon filter (Zeflour, 2 μm pore size) housed in a 47 mm PFA filter holder for 90 min. During the aerosol collection, cyclohexane (99.5%, Sigma-Aldrich) was introduced into the other chamber (photolysis chamber) to serve as an OH scavenger with an approximate mixing ratio of 10 ppm. After aerosol collection, the filter holder was removed from the SOA chamber and connected to the photolysis chamber. The collected aerosol was vaporized and introduced into the photolysis chamber by flowing heated zero air (which was heated by applying a heat gun to a 15 cm section of 1/4 in. clean stainless steel tubing located upstream of the filter) through the filter for 60 min. This process resulted in a rapid increase in various organic nitrate signals (*t* = −145 to −85 min; red shaded in Figure 1). After the signals reached the maximum levels at the end of the injection period, slight decays were observed during the dark period, most likely due to loss of vapors to the chamber wall.^28–31^ We monitored these slight decays for at least an hour to constrain the vapor wall loss rates of gaseous organic nitrates.^19^ In this study, we assumed that vapor wall loss follows first-order kinetics based on our previous study on synthetic hydroxy nitrates, where their measured decay behaviors were explained well by first-order rate constants on the time scale of the photolysis experiments. Then, UV lights were turned on to initiate the photolysis reaction, marking the beginning of the photolysis experiments. The injected cyclohexane scavenged OH radicals potentially generated from the chamber background (wall)^32^ and photolysis of organic hydroperoxides evaporated from the SOA on the filter. Note that it is possible that gaseous organic nitrates lost to the wall partition back to the gas phase during their photolytic loss or due to the slightly increased chamber temperature when the lights were on, potentially acting as a source of organic nitrates in the photolysis chamber. The UV irradiation continued for 3 h (*t* = 0 to 180 min; blue shaded in Figure 1). We then kept the measurements going for another hour and a half after the UV irradiation was turned off to again monitor and constrain the decay of organic nitrates under dark conditions (*t* = 180 to 270 min; gray shaded in Figure 1).

**Figure 1:**
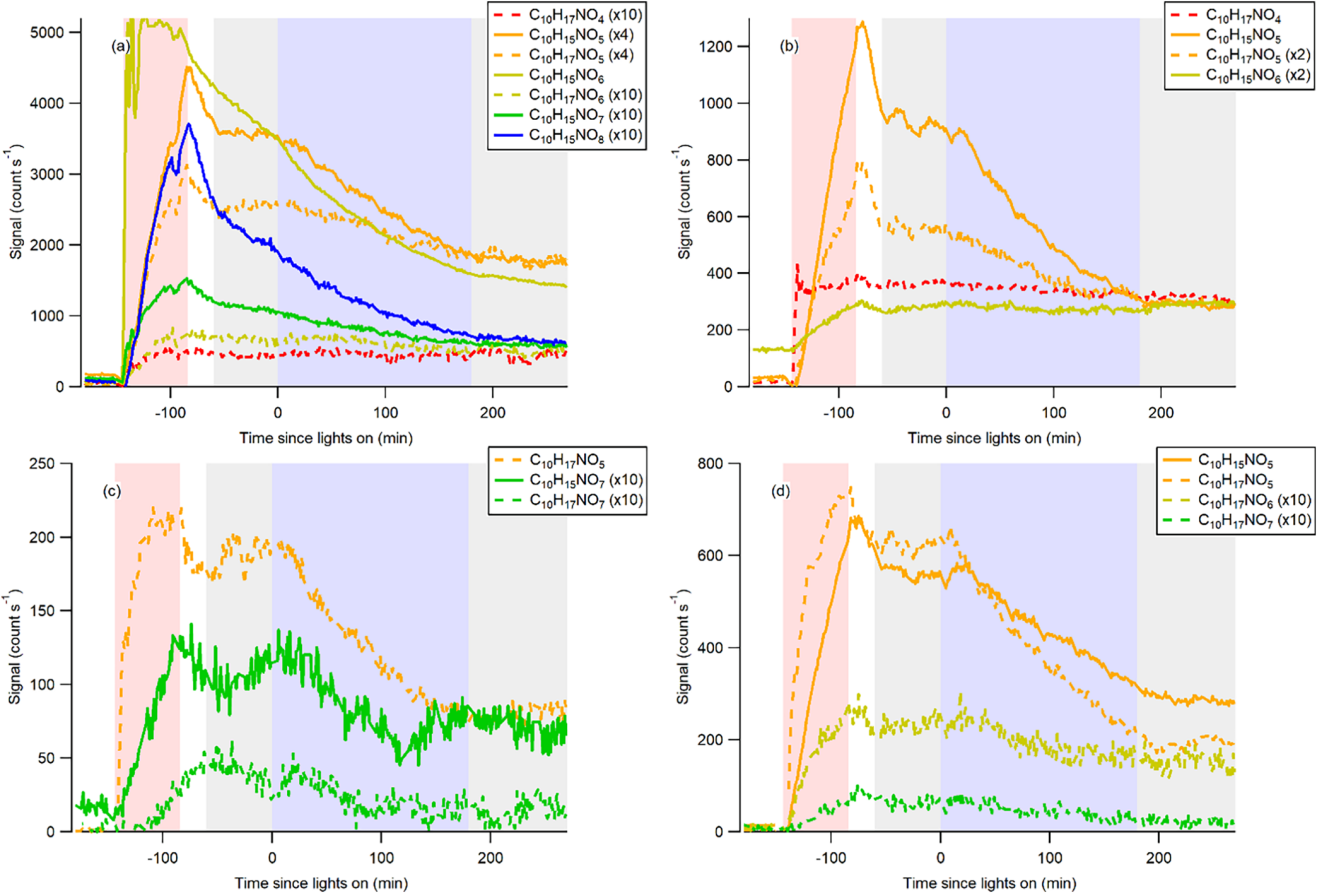
HR-ToF-CIMS time-series of C_10_ gaseous monoterpene organic nitrates showing photolysis decay (Table 1): (a) OH radical oxidation of α-pinene in the presence of NO, (b) OH radical oxidation of β-pinene in the presence of NO, (c) NO_3_ radical oxidation of α-pinene, and (d) NO_3_ radical oxidation of β-pinene. Red shaded color refers to the injection period, gray shaded color represents the dark period, and purple shaded color represents the UV irradiation period.

### Instrumentation

A high-resolution time-of-flight chemical mass spectrometer (HR-ToF-CIMS; Aerodyne Research Inc.) was used to monitor the gas-phase organic species, including gaseous organic nitrates, in the photolysis chamber.^19,27^ Reagent ions were generated by flowing a mixture of methyl iodide and dry N_2_ (Airgas) through a ^210^Po source (P-2021; NRD Inc.). The sampling rate through the Teflon tubing from the chamber was 3 L min^–1^, which was the sum of HR-ToF-CIMS (1.7 L min^–1^) and excess flow going to the exhaust (1.3 L min^–1^). Gas-phase background was taken for 1 min every 30 min of measurements by overflowing dry N_2_ at 3.5 L min^–1^. Another vacuum pump immediately upstream of the background dry N_2_ line pulled air at 6.5 L min^–1^ to avoid backflushing of dry N_2_ into the chamber and to keep the sampling flow rate constant (3 L min^–1^). Detected molecular formulas presented in this study are of iodide adducts unless otherwise noted. The data were preaveraged to 10-s and were analyzed using Tofware v2.5.11 in Igor Pro 6.37. NO and NO_2_ concentrations were measured by using a NO_*x*_ analyzer (Thermo 42C) and cavity attenuated phase shift spectroscopy (CAPS; Aerodyne Research Inc.), respectively. The small increases in NO_*x*_ levels during photolysis experiments and blank experiments (only cyclohexane was injected prior to UV irradiation) are comparable (Table S2). This is possibly due to the presence of background NO_*z*_ (reactive oxidized nitrogen species excluding NO_*x*_) contaminants in the chamber and the fact that the concentrations of total gaseous organic nitrates introduced were relatively low.

### Calculation of Photolysis Rate Constants in Chamber and Ambient Conditions

The HR-ToF-CIMS monitored the decay trends of gas-phase organic nitrates represented by individual molecular formulas. Accordingly, the detected signal is not isomer-resolved and reflects the summed contribution of all structural isomers sharing the same elemental composition. Their loss processes during chamber experiments include vapor wall loss, photolysis, OH radical oxidation, ozonolysis, and NO_3_ radical oxidation. Vapor wall loss describes a process in which gaseous molecules are absorbed into the chamber surface layer and slowly diffuse into the inner layer,^31^ acting as a sink of gaseous molecules. This loss process takes place during both dark and irradiation periods. On the other hand, photolysis occurs only during the irradiation period. OH radical oxidation was considered negligible throughout the experiment in this study, given the introduction of ∼10 ppm of cyclohexane as an OH scavenger. Upon the beginning of photolysis, NO_2_ could be released from photolabile gaseous organic nitrates and produce a small amount of ozone and NO_3_ radical.^19^ Most of the oxidation products of α-pinene and β-pinene were expected to no longer possess a double bond because the initial oxidation reaction most likely attacked the only double bond present in these precursor compounds, though a minor fraction might proceed via hydrogen abstraction, allowing the double bond to be retained.^33^ Based on the measured NO_*x*_ levels during the irradiation period (Table S2) and subsequent dark period, the expected levels of O_3_ (based on the titrated amount of NO) and NO_3_ in the photolysis chamber were <0.4 ppb and <1 × 10^–3^ ppt, respectively. Using published ozonolysis and NO_3_ radical oxidation rate constants for α-pinene and β-pinene,^2,34^ their combined contribution to the measured decay rates is <5% on average. Negligible contributions of ozonolysis and NO_3_ radical oxidation are also evident by the observation that the decay rates in the dark (*t* = 180 to 270 min) following the lights on periods are not faster than those during the dark period (*t* = −60 to 0 min) prior to lights on. Therefore, we considered that the observed decay trends of gas-phase organic nitrates were mostly governed by vapor wall loss and photolysis.

The irradiation spectra of light in the GTEC facility and that of sunlight (TUV, Atlanta) are not identical (Figure S1). In our previous study on photolysis of synthesized monoterpene organic nitrates,^19^ the ambient photolysis rate constants were derived using the chamber photolysis rate constants and the absorption cross sections measured for each synthesized monoterpene hydroxy nitrate standard. This approach is, however, not feasible for the complex mixtures of gaseous organic nitrates formed in situ from the oxidation of monoterpenes. To enable the conversion of chamber photolysis rate constants of gaseous organic nitrates to those in ambient conditions, we used the average ratio of the ambient to chamber photolysis rate constants (4.8 ± 2.1) of three different monoterpene hydroxy nitrates (α-pinene hydroxy nitrate, β-pinene hydroxy nitrate, and limonene hydroxy nitrate) determined in our previous study.^19^ This average ratio falls between that of α-nitrooxy acetone (5.9) and that of NO_2_ (4.0) (Table S3). It is noted that the same chamber facility and the same banks of blank lights were used in this study and our previous study. In addition, the conditions of black lights have been periodically monitored by estimating *j*_NO_2__, and no noticeable degradation was observed between this study period (2021) and our previous study (2019–2022).

## Results and Discussion

### Photolysis Rate Constants of Gaseous Organic Nitrates

The photolysis of gaseous organic nitrates formed from OH radical oxidation of α-pinene in the presence of NO, OH radical oxidation of β-pinene in the presence of NO, NO_3_ radical oxidation of α-pinene, and NO_3_ radical oxidation of β-pinene was studied in the GTEC facility. Duplicate experiments were performed for each of the systems investigated. The decay trends of gaseous monoterpene organic nitrates were monitored by HR-ToF-CIMS. Shown in Figure 2 is an example decay curve in log scale for the major gaseous organic nitrate (C_10_H_15_NO_5_) formed from NO_3_ radical oxidation of β-pinene. Since both vapor wall loss and photolysis follow first-order kinetics, the decay curve in log scale can be fitted via least-squares linear regression. While vapor wall loss was present during both the UV irradiation period and the dark period, photolysis occurred only during the UV irradiation period. Thus, the slope during the UV irradiation period represents the sum of the photolysis rate constant and vapor wall loss rate constant, whereas that during the dark periods represents the vapor wall loss rate constant only. Subtracting the decay rate constant during the dark periods (averaged) from that during the irradiation period, a photolysis rate constant for each of the gaseous organic nitrates can be obtained. The decay rates during the UV irradiation period and two dark periods are shown in Table S4.

**Figure 2:**
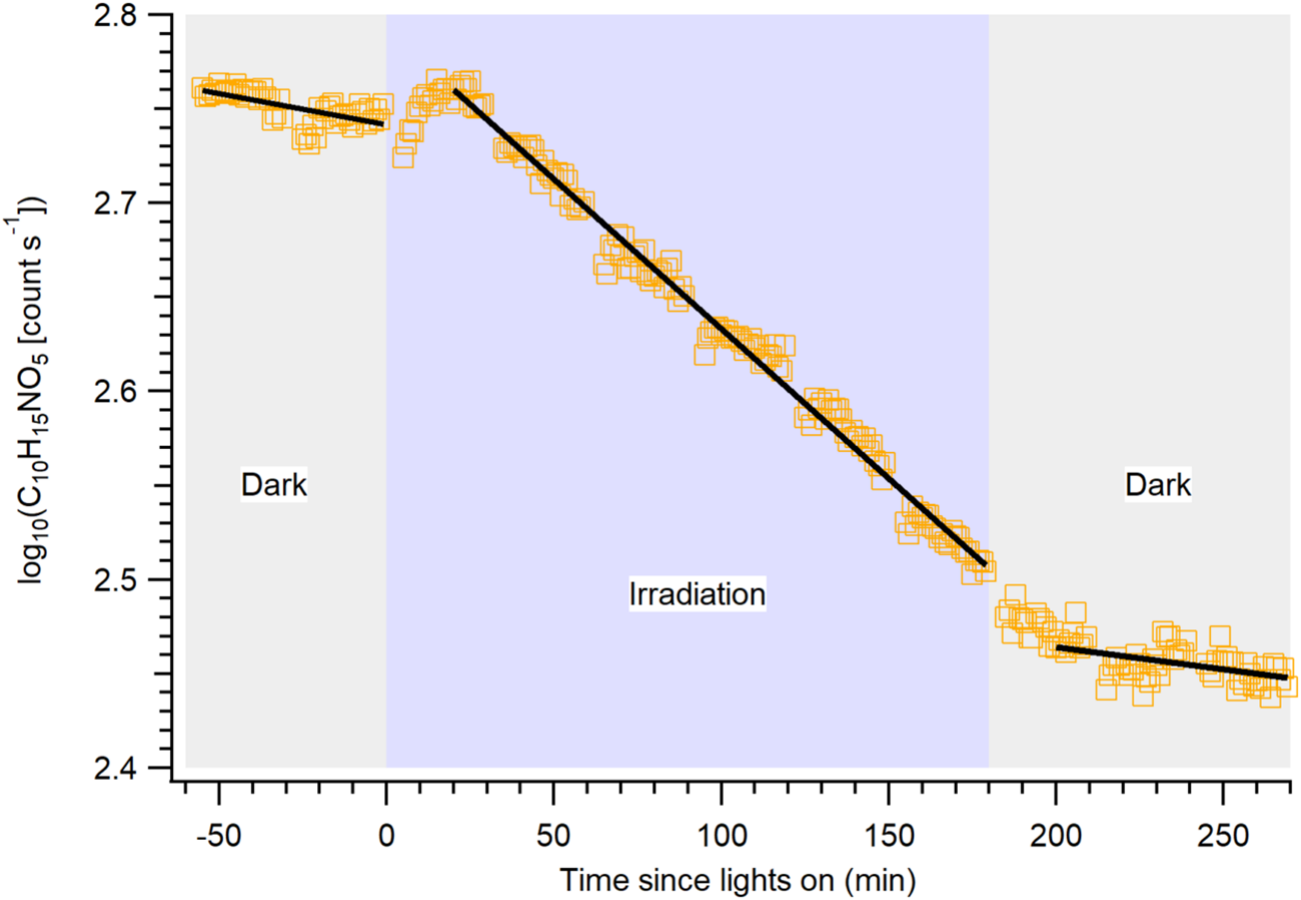
HR-ToF-CIMS time-series of gaseous monoterpene organic nitrate (C_10_H_15_NO_5_) formed from NO_3_ radical oxidation of β-pinene in log scale. Data points from *t* = −60 to 0 min, *t* = 20 to 180 min, and *t* = 200 to 270 min are used for least-squares linear regression fitting (black lines).

Table 1 shows the *j*_Chmbr_ values of C_10_ gaseous monoterpene organic nitrates formed from the OH radical oxidation of α-pinene in the presence of NO, the OH radical oxidation of β-pinene in the presence of NO, the NO_3_ radical oxidation of α-pinene, and the NO_3_ radical oxidation of β-pinene. Since monoterpenes contain ten carbons, C_10_ species are the main monoterpene organic nitrates. The organic nitrates in Table 1 were selected, as the decays in their signals during the irradiation period were higher than the dark periods. It is noted that while C_<10_ organic nitrates are also formed during monoterpene oxidation, their photolysis process can be confounded by their formation via photolysis of C_10_ organic nitrates. The photolysis of the C_10_ organic nitrates is determined to be as fast as 5.1 (C_10_H_15_NO_5_), 8.8 (C_10_H_15_NO_5_), 11.2 (C_10_H_17_NO_5_), and 13.4 (C_10_H_17_NO_5_) × 10^–5^ s^–1^, respectively. These rate constants are at least 2.2–24.4 times higher than synthetic monoterpene hydroxy nitrates investigated in the same GTEC facility,^19^ indicating the formation of a large suite of organic nitrates that are more prone to photolysis than synthetic hydroxy nitrates. Rapid photolysis of C_10_H_17_NO_5_ formed via NO_3_ radical oxidation of β-pinene, for example, is consistent with a proposed six-membered-ring structure bearing both a carbon–carbon double bond and a hydroperoxide group.^21^ Our previous work^19^ showed that the conjugation of carbon–carbon double bond and nitrooxy group increases absorption cross sections at 220–270 nm, yielding shorter photolysis lifetimes for synthetic monoterpene hydroxy nitrates compared to smaller alkyl nitrates. In addition, a hydroperoxide moiety acts as a chromophore that can further enhance absorption cross section and thus the photolysis rate.^35–37^ In the meanwhile, the chamber photolysis rate constants of organic nitrates with the same molecular formula (C_10_H_17_NO_4_) as the synthetic standards show comparable values (Figure S2). Some organic nitrates also exhibit no decay during the UV irradiation period, indicating that not all gaseous organic nitrates are susceptible to photolysis. It is important to note that the lack of observed decay does not necessarily mean the photolysis process is not important for a given gaseous organic nitrate. Since the gaseous non-nitrated organics and organic nitrates present during photolysis experiments were chemically diverse, it is possible that some specific organic nitrates were formed via photolysis of other organic nitrates, potentially canceling out the photolysis decay. Our previous study,^19^ for example, showed that photolysis of α-pinene, β-pinene, and limonene hydroxy nitrates (C_10_H_17_NO_4_) all led to the formation of C_10_H_17_NO_5_. More oxygenated organic nitrates could be more susceptible to this effect because further oxidation upon photolysis inevitably makes the compounds more oxidized. Therefore, little to no decay observed during the UV irradiation period for some organic nitrates may not necessarily indicate no photolysis, and this suggests that the measured chamber photolysis rate constants in this study can be considered to be lower limits. Overall, the chamber photolysis rate constants for photolabile organic nitrates range from 1.9–5.1, 0.9–8.8, 1.4–11.2, and 5.3–13.4 × 10^–5^ s^–1^ for gaseous monoterpene organic nitrates formed from OH radical oxidation of α-pinene in the presence of NO, OH radical oxidation of β-pinene in the presence of NO, NO_3_ radical oxidation of α-pinene, and NO_3_ radical oxidation of β-pinene, respectively (Table 1).

**Table 1:** Summary of Chamber Photolysis Rate Constants of C_10_ Gaseous Monoterpene Organic Nitrates^a^

	OH radical oxidation of α-pinene in the presence of NO	OH radical oxidation of β-pinene in the presence of NO	NO_3_ radical oxidation of α-pinene	NO_3_ radical oxidation of β-pinene
C_10_H_17_NO_4_	1.9 ± 0.9	0.9 ± 0.3	–	–
C_10_H_15_NO_5_	5.1 ± 0.6	8.8 ± 0.4	–	5.3 ± 0.3
C_10_H_17_NO_5_	4.2 ± 0.8	4.6 ± 0.6	11.2 ± 0.3	13.4 ± 2.5
C_10_H_15_NO_6_	2.6 ± 0.6	2.4 ± 0.2	–	–
C_10_H_17_NO_6_	2.7 ± 1.5	–	–	5.9 ± 0.5
C_10_H_15_NO_7_	2.9 ± 0.1	–	4.9 ± 2.0	–
C_10_H_17_NO_7_	–	–	1.4 ± 0.0	6.7 ± 11.8
C_10_H_15_NO_8_	3.3 ± 0.5	–	–	–

a Values are averages and standard deviations of duplicate experiments, expressed in 10^–5^ s^–1^. Values are not provided for organic nitrates exhibiting increases in signals during the UV irradiation period (shown as –).

The photolysis rate constant depends on actinic flux, absorption cross section, and quantum yield, which are all functions of the wavelength. Despite the similar magnitude, the distribution of actinic flux over the interested wavelengths in the GTEC facility and in typical ambient conditions during summer in Atlanta is not quite comparable (Figure S1). Without knowledge of the absorption cross section, there is no direct means to estimate the ambient photolysis rate constants from the measured chamber photolysis rate constants. Based on the scaling factor derived from monoterpene hydroxy nitrates as surrogate compounds (Table S3),^19^ the ambient photolysis rate constants range from 9.1–24.6 (an equivalent lifetime of 1.1–3.0 h), 4.4–42.2 (0.7–6.4 h), 6.9–53.5 (0.5–4.0 h), and 25.2–64.3 (0.3–1.1 h) × 10^–5^ s^–1^ for gaseous monoterpene organic nitrates formed from OH radical oxidation of α-pinene in the presence of NO, OH radical oxidation of β-pinene in the presence of NO, NO_3_ radical oxidation of α-pinene, and NO_3_ radical oxidation of β-pinene, respectively. Photolysis rate constants of mononitrates, dinitrates, and multifunctional nitrates have been reported, covering more than 3 orders of magnitude (Figure 3).^12,14–17,19,38^ The rate constants measured in this study are on the upper end, indicating that gaseous monoterpene organic nitrates, on average, undergo rapid photolysis.

**Figure 3:**
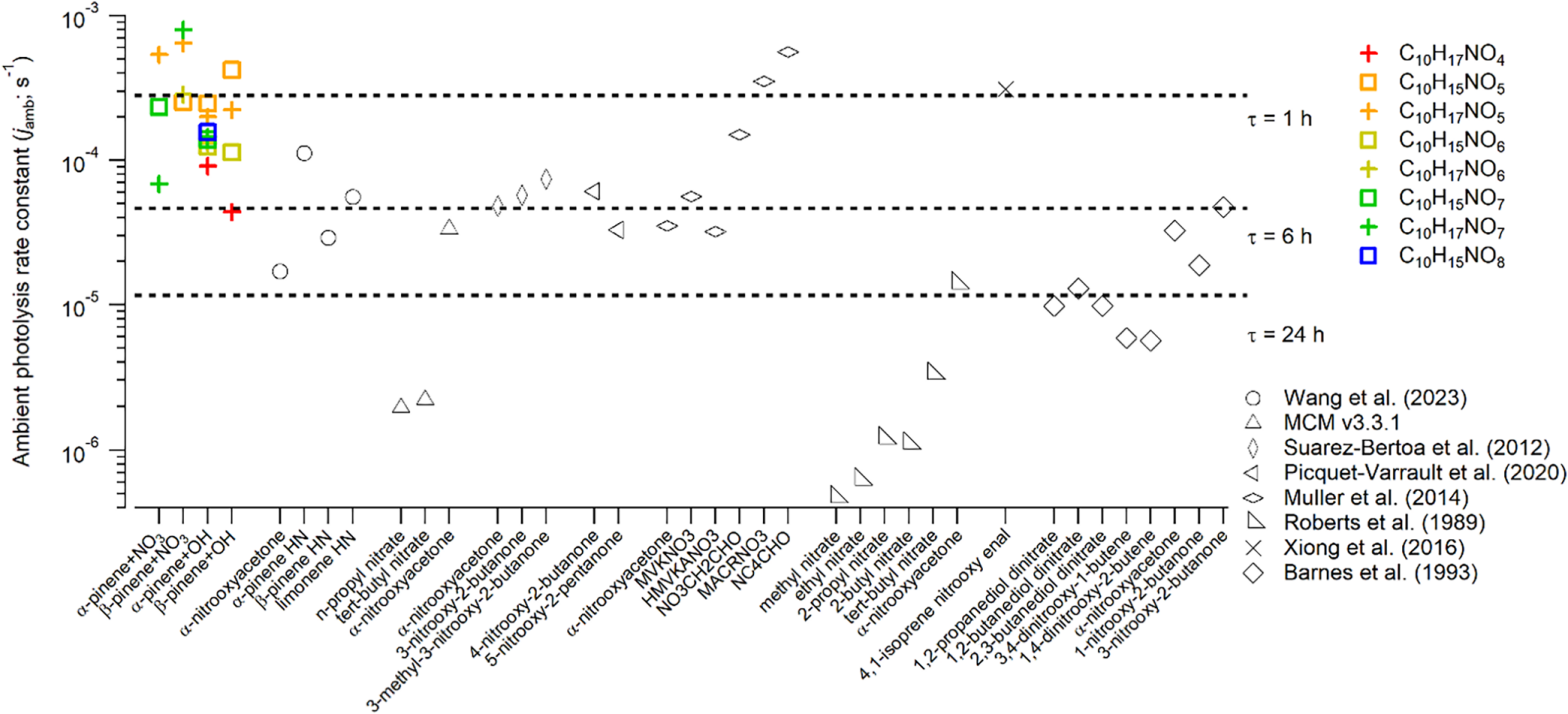
Comparison of ambient photolysis rate constants between this study and the literature.

It is worth noting that these extrapolated ambient photolysis rate constants are subject to additional uncertainty related to the use of a simple scalar scaling factor. The influence of adjacent functional groups on photolysis of nitrate functional groups can vary due to differences in their absorption cross sections and their synergy with nitrate groups. The scaling factor is derived from synthetic monoterpene nitrates with hydroxy groups, which would contribute differently to photolysis than other chromophores, such as hydroperoxides and carbonyls.^35,39^ Given the variety of functional groups likely present on the measured monoterpene organic nitrates, the scaling factor must be adjusted when possible.

### Evaluating Chemical Structures of Gaseous Organic Nitrates Based on Photolysis Rate Constants

We combined our measurements of the photolysis rate constants with the understanding about factors affecting the photolysis rate constants in the literature to gain new insights into the expected chemical structures of monoterpene organic nitrates. From previous studies, it has been reported that the photolysis rate constants of organic nitrates greatly vary, depending on the presence and proximity of additional functional groups^15–17^ and the carbon backbone.^19,40^ The presence of chromophores (e.g., carbonyl functional groups, conjugated double bond, etc.) adjacent to a nitrate functional group provides a synergistic enhancement in absorption cross sections, leading to greater photolysis frequencies of organic nitrates than those without chromophores.^14,17^ Picquet-Varrault et al.^16^ found that the effects of chromophores decreased as they were further away from the nitrate functional group. Roberts and Fajer^38^ reported that the presence of another commonly found functional group in gaseous oxidation products, the hydroxy functional group, led to a decrease in absorption cross sections. For instance, compared to ethyl nitrate, nitrooxy ethanol showed a factor of >5 lower absorption cross section at a wavelength of 300 nm. On the other hand, the absorption cross sections of monoalkyl nitrates increased with a longer carbon chain across the measured wavelengths.^40^ Greater photolysis frequencies observed for gaseous organic nitrates formed from monoterpene oxidation can result from the presence and proximity of additional functional groups and/or the greater number of carbon atoms.

*j*_Chmbr_ varies depending not only on the molecular formulas of organic nitrates but also on the type of precursor VOCs and oxidation conditions. For instance, the C_10_H_15_NO_5_ formed from OH radical oxidation of α-pinene and β-pinene in the presence of NO and NO_3_ radical oxidation of β-pinene shows a noticeable photolysis decay, though the organic nitrate with the same molecular formula formed from NO_3_ radical oxidation of α-pinene does not exhibit any decrease during the irradiation period compared to during the dark period. The rapid photolysis of C_10_H_15_NO_5_ formed from NO_3_ radical oxidation of β-pinene is consistent with the proposed chemical structure bearing a nitrate functional group at the α position of a carbonyl group, i.e., dicarbonyl nitrate.^21^ The relatively higher photolysis rate constants of gaseous organic nitrates formed from NO_3_ radical oxidation of β-pinene than from the other systems (Table 1) may be also explained by the decomposition of initially abundant β-nitrooxyalkoxy radical, breaking the six-membered ring and forming a carbonyl functional group adjacent to a nitrate functional group. Although the final chemical structures may differ depending on subsequent chemistry, a nitrooxy group is often present at the α position of a carbonyl group. On the other hand, alkoxy radical bond scissions are favored during NO_3_ radical oxidation of α-pinene,^41^ resulting in negligible formation of organic nitrates with a nitrooxy group being present at the α position of a carbonyl group. This highlights how differences in chemical structures can affect the photolability of organic nitrates, despite having the same molecular formula.

### Proposed Photolysis Mechanism of Organic Nitrates Formed from NO_3_ Radical Oxidation of β-Pinene

Unlike previous studies using synthetic standards,^16,19^ the exact chemical structures of many organic nitrates formed from OH and NO_3_ oxidation of monoterpenes, as in this study, are either unknown or highly uncertain. Here, we chose the NO_3_ radical oxidation of β-pinene system to study the photolysis mechanism because our measured photolysis rate constants are consistent with the proposed chemical structures in Claflin and Ziemann,^21^ as discussed in the previous section.

A wide variety of molecular formulas are detected by the HR-ToF-CIMS, including both non-nitrated organics and organic nitrates. Based on the major products detected, the photolysis mechanisms of C_10_H_15_NO_5_ involving photolysis of both nitrate and carbonyl functional groups are proposed, as shown in Figure 4. Muller et al.^17^ discussed that photolysis frequencies of certain isoprene nitrates were enhanced owing to the presence of an adjacent carbonyl group and that the likely major photolysis channel was the dissociation of the O–NO_2_ bond. On the other hand, Picquet-Varrault et al.^16^ reported that the dominant photolysis pathway was the dissociation of the C­(O)–C bond for 4-nitrooxy-2-butanoone, confirmed by the measurement of a close to unity yield of peroxyacetyl nitrate. Together, these studies suggested that photolysis of gaseous organic nitrates does not always involve the dissociation of the O–NO_2_ bond but can also lead to the breakdown of the C­(O)–C bond. In this work, the dissociations of both nitrooxy and carbonyl groups likely occur because photolysis products from both channels are observed to be formed during the irradiation period. For instance, photolysis of the nitrooxy group in C_10_H_15_NO_5_ produces C_8–10_ non-nitrated organics and organic nitrates, whereas photolysis of the carbonyl group often produces smaller compounds with an intact nitrooxy group, such as CHNO_4_, C_2_H_3_NO_5_, and C_3_H_5_NO_4_ (Figures 4 and S3). While the Norrish type I reaction appears to be responsible for the formation of C_1–2_ organic nitrates, the Norrish type II reaction contributes to the formation of C_3_H_5_NO_4_ as well as the formation of organic nitrates with the same molecular formula as that of the starting compound (C_10_H_15_NO_5_). Overall, the signal abundance from larger C_8–10_ non-nitrated organics and organic nitrates outweighs the sum of signals from smaller compounds with an intact nitrooxy group, suggesting that the dissociation of the O–NO_2_ bond may be the major photolysis pathway.

**Figure 4:**
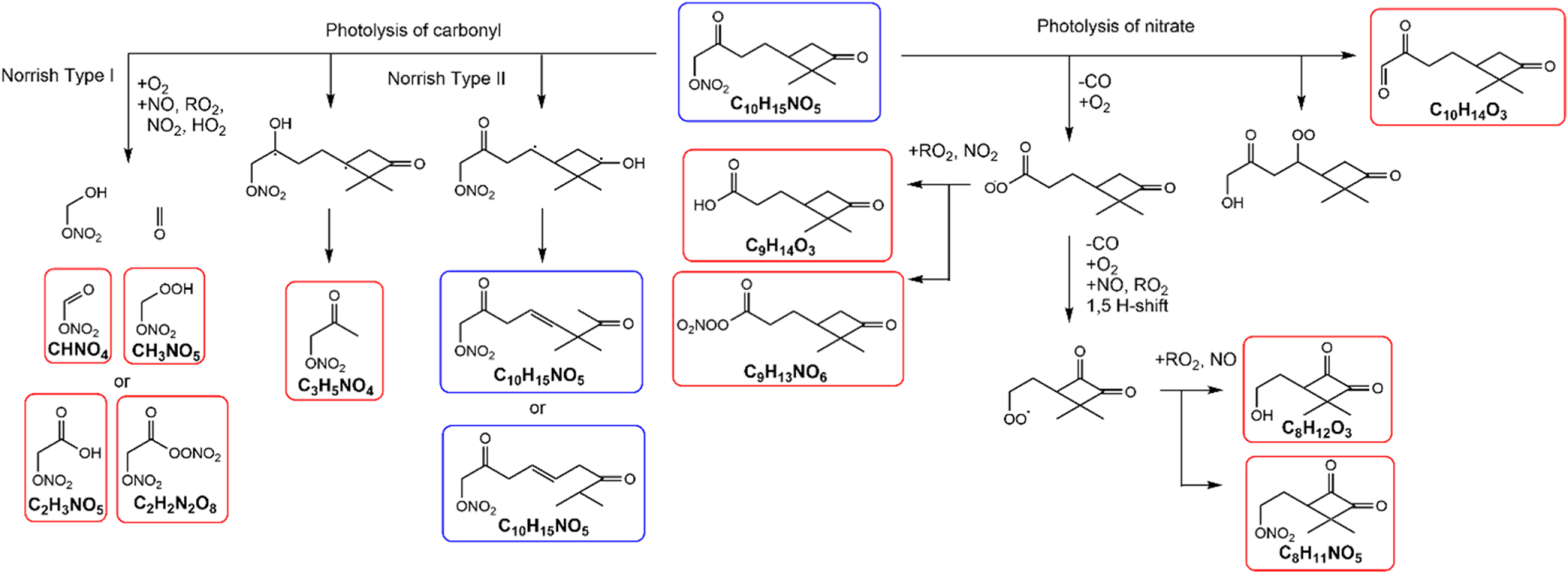
Proposed photolysis mechanisms of C_10_H_15_NO_5_ formed from NO_3_ radical oxidation of β-pinene, forming various gas-phase products detected by the HR-ToF-CIMS (boxed in red) during photolysis experiments. Chemicals boxed in blue share the same chemical formula as the starting compound (C_10_H_15_NO_5_), to indicate that one of the photolysis pathways can produce compounds with the same chemical formula.

## Atmospheric Implications

In this study, we investigated the photolysis of gaseous monoterpene organic nitrates using a novel dual chamber approach in which organic nitrates formed in one chamber were first collected onto a filter and then introduced into another chamber through evaporation. We found that photolysis rate constants of gaseous organic nitrates formed from OH and NO_3_ radical oxidation of monoterpenes are either comparable or higher than organic nitrates previously reported, with atmospheric lifetimes of as short as 0.43 ± 0.20 h (Figure 3). This is an important finding, as the photolysis fate of gaseous monoterpene organic nitrates has not been historically considered as an important loss mechanism. Despite the ubiquitous nature and abundance of monoterpene organic nitrates, experimentally constrained data on their photolysis fate are limited – with only one study investigating the photolysis of synthesized monoterpene organic nitrates thus far.^19^ In particular, owing to the high uncertainty in deducing the exact chemical structures of various organic nitrates as well as the lack of commercial availability of a wide variety of organic nitrates, photolysis studies on in situ generated organic nitrates formed from monoterpene oxidation are nonexistent. The novel approach taken in this study may be useful for studying the photolysis fate of gaseous organic nitrates formed from other types of precursor VOCs.

To understand the relative importance of the photolysis fate for gaseous organic nitrates, their lifetimes against photolysis, OH radical oxidation, and dry deposition are compared (Table 2). As with the case of the proposed photolysis mechanism, we focused on gaseous organic nitrates (C_10_H_15_NO_5_, C_10_H_17_NO_5_, and C_10_H_17_NO_6_) formed from NO_3_ radical oxidation of β-pinene, with known chemical structures proposed in Claflin and Ziemann.^21^ To calculate the lifetime against OH radical oxidation, the structure–activity relationships (SARs)^42–44^ were used to estimate the OH radical oxidation rate constant for each organic nitrate. Assuming an average OH radical concentration of 1.5 × 10^6^ molecules cm^–3^, OH radical oxidation lifetimes are 10.8, 17.7, and 4.3 h for C_10_H_15_NO_5_, C_10_H_17_NO_5_, and C_10_H_17_NO_6_, respectively. Note that depending on the actual OH radical concentration, their lifetimes against OH radical oxidation could either increase or decrease. Dry deposition lifetimes are calculated as the ratio of dry deposition velocity over a boundary layer height. Using the measured boundary layer height during the Southern Oxidant and Aerosol Study (SOAS) campaign (375–1300 m)^6^ and the measured dry deposition velocity of organic nitrates (1–1.5 cm s^–1^),^45^ dry deposition lifetimes are found to be 7–36 h. Overall, photolysis appears to be the dominant fate for C_10_H_15_NO_5_ and C_10_H_17_NO_5_, while OH radical oxidation can be a competitive loss mechanism with photolysis for C_10_H_17_NO_6_.

**Table 2:** Lifetimes (Inverses of Rate Constants) against Photolysis, OH Radical Oxidation, and Dry Deposition for Gaseous Organic Nitrates (C_10_H_15_NO_5_, C_10_H_17_NO_5_, and C_10_H_17_NO_6_) Formed from NO_3_ Radical Oxidation of β-Pinene^a^

	photolysis (h)	OH radical oxidation (h)	dry deposition (h)
C_10_H_15_NO_5_	1.1	10.8	7–36
C_10_H_17_NO_5_	0.4	17.7	7–36
C_10_H_17_NO_6_	1.0	4.3	7–36

a Lifetimes against OH radical oxidation are estimated based on structure-activity relationships (SARs)^42,43,55^ with an OH concentration of 1.5 × 10^6^ molecules cm^–3^. Dry deposition lifetimes are estimated as a ratio of boundary layer height (375–1300 m during the SOAS campaign^6^) over dry deposition velocity (1–1.5 cm s^–1^).^45^

The treatment of the photolysis process for gaseous monoterpene organic nitrates in regional and global chemical transport models may need to be updated. In previous studies using chemical transport models, the photolysis rate constant for monoterpene nitrates was represented by those of n-propyl nitrate (i.e., ∼10^–6^ s^–1^),^46^ those of *tert*-butyl nitrate,^47^ or methyl hydroperoxide.^48^ The photolysis rate constants of these surrogate compounds are a few orders of magnitude lower than those found in this study. The incorporation of fast photolysis of gaseous organic nitrates formed from monoterpene oxidation could alter the organic nitrate life cycle, dynamics of NO_*x*_ recycling, and ozone formation. In particular, the role of monoterpene organic nitrates in NO_*x*_ recycling as well as transporting NO_*x*_ over a regional scale could be enhanced if rapid photolysis could act as a source of NO_*x*_. While hydrolysis has been assumed to be the major fate of monoterpene organic nitrates in models,^49–51^ it has been shown previously from laboratory chamber experiments and molecular structure perspectives that not all monoterpene organic nitrates undergo hydrolysis.^20,27,52,53^ Gas-phase photolysis of monoterpene organic nitrates could be an important missing loss process to account for the observed short lifetimes of atmospheric organic nitrates in both gas and particle phases.^7,54^ With the emergence of the recent experimental studies investigating the fates of monoterpene organic nitrates, models should be better constrained to more accurately evaluate the impacts of monoterpene organic nitrates on NO_*x*_ cycling and ozone formation.

## Supplementary Material



## Data Availability

The chamber experiment data used in this study are available in the Index of Chamber Atmospheric Research in the United States (ICARUS) database, https://icarus.ucdavis.edu/experimentset/273.

## Notes

Tables S1–S4 and Figures S1–S3 referenced in the main text, providing additional methodological details, expanded results, and supplementary analyses (PDF)

## ⊥

Department of Mechanical Engineering, University of Colorado Boulder, Boulder, Colorado 80309, United States
