# Supplementary material for: Rapid Photolysis of Gaseous Organic Nitrates Formed from Hydroxyl and Nitrate Radical Oxidation of α‑Pinene and β‑Pinene
Source: ACS EST Air. 2025 Oct 7;2(11):2445–54. doi: 10.1021/acsestair.5c00183 (PMC12624529; doi:10.1021/acsestair.5c00183)
Supplement: Supplementary file 1 [file ea5c00183_si_001.pdf]

Supporting Information for

Rapid photolysis of gaseous organic nitrates formed from hydroxyl and nitrate radical oxidation of α-pinene and β-pinene

Masayuki Takeuchi[a#], Yuchen Wang[bc], Nga L. Ng[acd*]

[a] School of Civil and Environmental Engineering, Georgia Institute of Technology, Atlanta, Georgia, 30332, USA
[b] College of Environmental Science and Engineering, Hunan University, Hunan, 410082, China
[c] School of Chemical and Bimolecular Engineering, Georgia Institute of Technology, Atlanta, Georgia, 30332, USA
[d] School of Earth and Atmospheric Sciences, Georgia Institute of Technology, Atlanta, Georgia, 30332, USA
[*] Email: ng@chbe.gatech.edu

Present address:
[#] M.T.: Department of Mechanical Engineering, University of Colorado Boulder, Boulder, Colorado, 80309, USA

Table S1. Summary of the initial concentrations of VOC and oxidants used to form SOA. Values in parenthesis for NO indicate the continuous injection of NO during oxidation reaction.

Type of experiment	VOC (ppm)	H_2O_2 (ppm)	NO (ppb)	N_2O_5 (ppb)
OH radical oxidation of α-pinene in the presence of NO	3	9	500 (+ 10 min^{-1})	-
OH radical oxidation of β-pinene in the presence of NO	1.2	9	500 (+ 10 min^{-1})	-
NO_3 radical oxidation of α-pinene	3	-	-	800
NO_3 radical oxidation of β-pinene	3	-	-	320

Table S2. Increases in NO_x concentrations between the beginning and end of UV irradiation period. Values after ± indicates one standard deviation of duplicate photolysis experiments and of triplicate blank experiments.

Type of experiment	ΔNO (ppb)	$ΔNO_2$ (ppb)	$ΔNO_x$ (ppb)
OH radical oxidation of α-pinene in the presence of NO	0.41 ± 0.01	0.00 ± 0.28	0.41 ± 0.28
OH radical oxidation of β-pinene in the presence of NO	0.29 ± 0.00	0.49 ± 0.01	0.78 ± 0.01
NO_3 radical oxidation of α-pinene	0.24 ± 0.18	0.28 ± 0.02	0.51 ± 0.20
NO_3 radical oxidation of β-pinene	0.38 ± 0.05	0.60 ± 0.06	0.97 ± 0.01
Blank	0.49 ± 0.10	0.54 ± 0.37	1.04 ± 0.43

Table S3. Chamber and ambient photolysis rate constants of α-pinene hydroxy nitrate, β-pinene hydroxy nitrate, limonene hydroxy nitrate, and α-nitrooxyacetone reported in Wang, et al. [1] as well as NO_2.

	j_{Chmbr} (10^{-5} s^{-1})	j_{Amb} (10^{-5} s^{-1})[a]	j_{Amb} / j_{Chmbr}
α-pinene hydroxy nitrate	2.3	8.3–14	3.6–6.1
β-pinene hydroxy nitrate	0.55	1.7–4.1	3.1–7.5
limonene hydroxy nitrate	1.3	3.0–8.1	2.3–6.2
α-nitrooxyacetone	0.29	1.7	5.9
NO_2	224	892	4.0

[a] The lower and upper bounds correspond to values obtained from using the average quantum yield and wavelength-dependent quantum yield, respectively.

Table S4. Decay rates of the C_{10} gaseous monoterpene organic nitrates reported in Table 1 during the UV irradiation period (k_UV in s^{-1}), two dark periods (k_dark1 and k_dark2 in s^{-1}), and the average of the two dark periods (k_dark in s^{-1}).

Type of experiment	Compound	k_UV	k_dark	k_dark1	k_dark2
OH radical oxidation of α-pinene in the presence of NO	$C_{10}H_{17}NO_4$	5.38E-08	-1.90E-05	-4.69E-05	8.89E-06
	$C_{10}H_{15}NO_5$	5.94E-05	8.04E-06	5.96E-06	1.01E-05
	$C_{10}H_{17}NO_5$	3.37E-05	-7.82E-06	-2.47E-05	9.06E-06
	$C_{10}H_{15}NO_6$	6.57E-05	3.98E-05	5.74E-05	2.23E-05
	$C_{10}H_{17}NO_6$	2.28E-05	-4.48E-06	-3.09E-05	2.20E-05
	$C_{10}H_{15}NO_7$	4.63E-05	1.73E-05	3.19E-05	2.74E-06
	$C_{10}H_{15}NO_8$	8.81E-05	5.55E-05	8.29E-05	2.80E-05
OH radical oxidation of β-pinene in the presence of NO	$C_{10}H_{17}NO_4$	1.44E-05	5.24E-06	-7.11E-06	1.76E-05
	$C_{10}H_{15}NO_5$	9.37E-05	5.71E-06	-2.40E-06	1.38E-05
	$C_{10}H_{17}NO_5$	4.82E-05	1.70E-06	-5.30E-06	8.71E-06
	$C_{10}H_{15}NO_6$	7.18E-06	-1.65E-05	-3.08E-05	-2.22E-06
NO_3 radical oxidation of α-pinene	$C_{10}H_{17}NO_5$	8.65E-05	-2.51E-05	-2.60E-05	-2.42E-05
	$C_{10}H_{15}NO_7$	4.16E-05	-6.93E-06	-3.80E-05	2.42E-05
	$C_{10}H_{17}NO_7$	1.04E-04	9.01E-05	9.01E-05	N/A
NO_3 radical oxidation of β-pinene	$C_{10}H_{15}NO_5$	5.85E-05	5.88E-06	8.62E-06	3.13E-06
	$C_{10}H_{17}NO_5$	1.11E-04	-2.30E-05	-1.18E-05	-3.42E-05
	$C_{10}H_{17}NO_6$	3.82E-05	-2.13E-05	-4.23E-05	-3.04E-07
	$C_{10}H_{17}NO_7$	1.05E-04	-6.14E-05	-4.53E-05	-7.76E-05

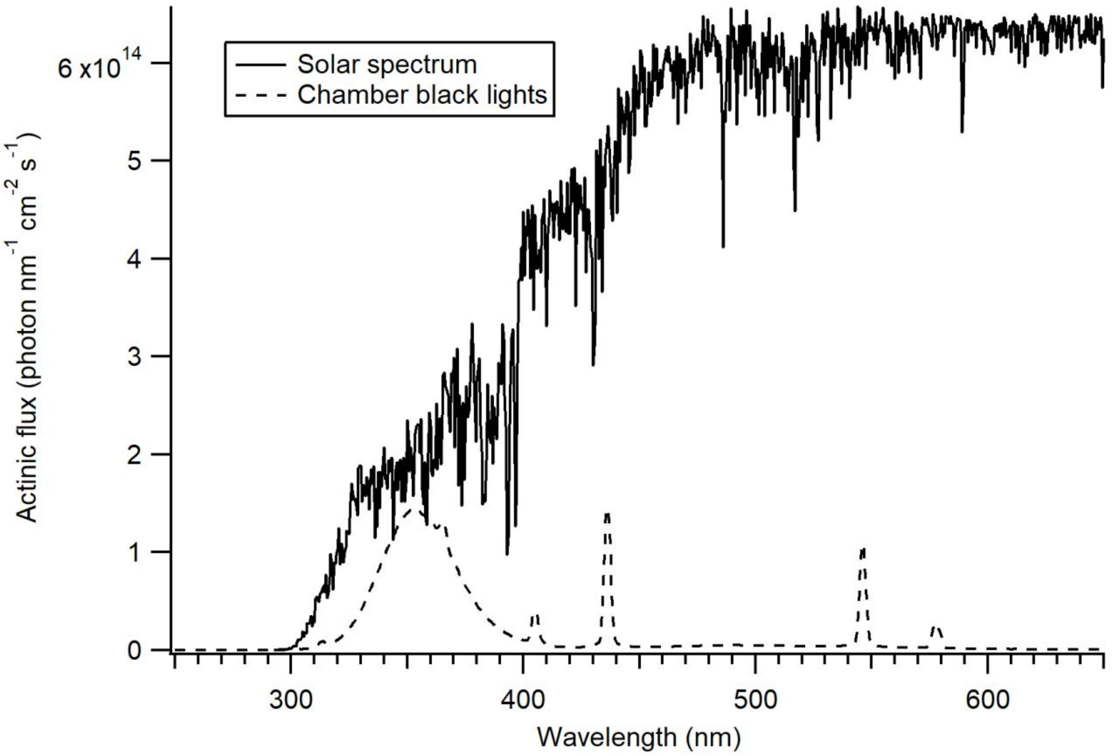

Figure S1. Comparison of actinic fluxes. The dashed line represents the actinic flux of black lights in the GTEC facility, while the solid line is the solar actinic flux calculated at 12:00 solar time, August 1 at 33.75° latitude North, overhead ozone column 300 Du, and albedo 0.1 (TUV NCAR).

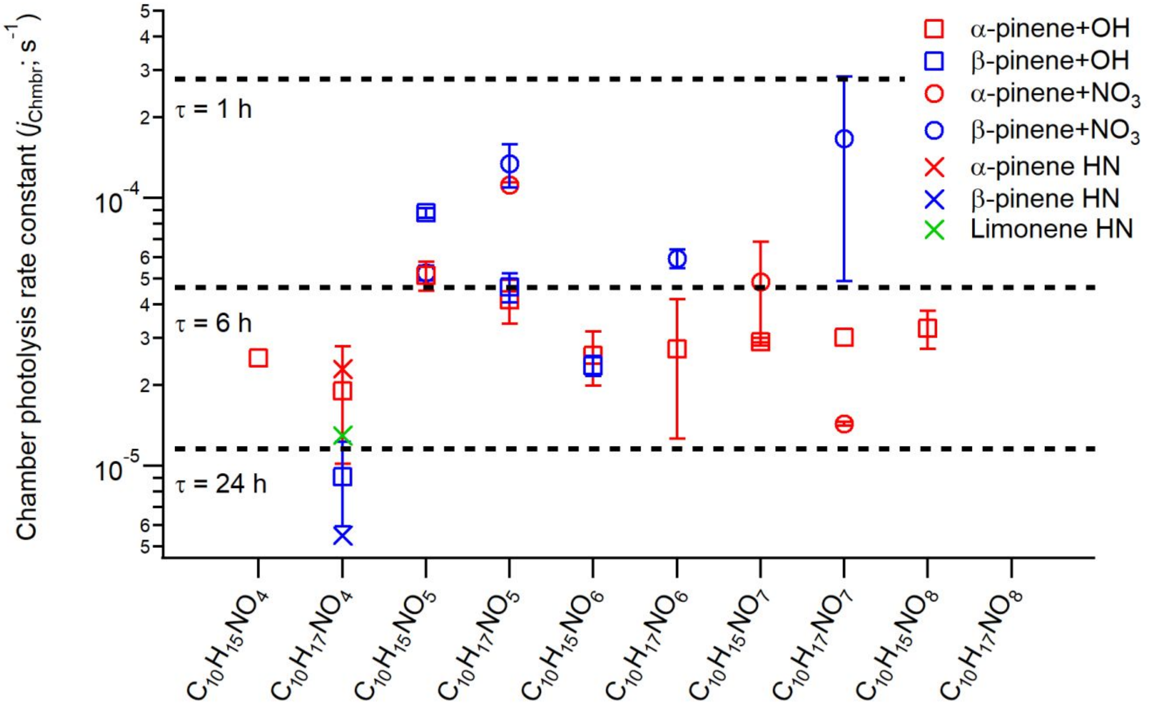

Figure S2. Comparison of chamber photolysis rate constants of gaseous monoterpene organic nitrates. Markers and error bars represent averages and standard deviations of duplicate experiments. Values for α-pinene hydroxy nitrate (HN), β-pinene HN, and limonene HN are from our previous study.[1]

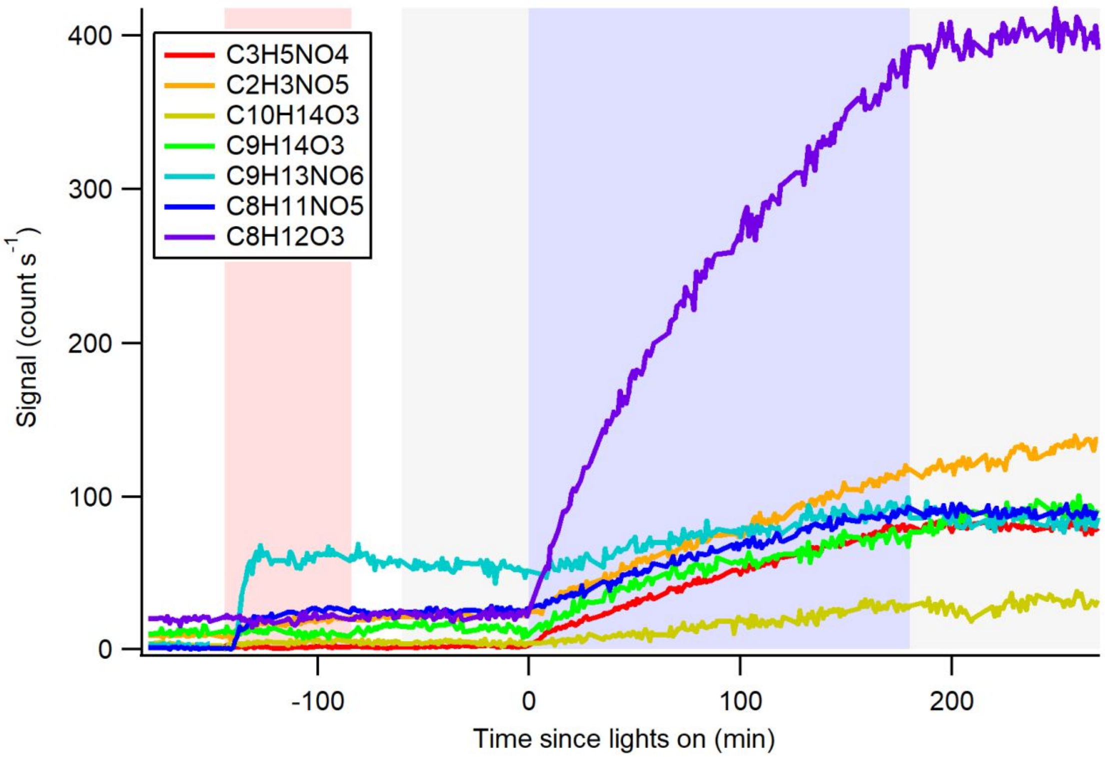

Figure S3. HR-ToF-CIMS time-series of gas-phase products (as shown in Figure 4 in the main text) formed from the photolysis of $C_{10}H_{15}NO_5$, a major organic nitrate generated from NO_3 radical oxidation of β-pinene. Red shaded color refers to the injection period, grey shaded color represents the dark period, and purple shaded color represents the UV irradiation period.

References

1. Wang, Y. C.; Takeuchi, M.; Wang, S. Y.; Nizkorodov, S. A.; France, S.; Eris, G.; Ng, N. L., Photolysis of Gas-Phase Atmospherically Relevant Monoterpene- Derived Organic Nitrates. *J Phys Chem A* **2023**.
